# Combination bronchodilator therapy in the management of chronic obstructive pulmonary disease

**DOI:** 10.1186/1465-9921-14-49

**Published:** 2013-05-08

**Authors:** Donald P Tashkin, Gary T Ferguson

**Affiliations:** 1 Department of Medicine, David Geffen School of Medicine at UCLA, 405 Hilgard Avenue, Los Angeles, CA, 90095, USA; 2 Pulmonary Research Institute of Southeast Michigan, 28815 Eight Mile Road, Suite 103, Livonia, MI, 48152, USA

**Keywords:** COPD, Combination therapy, Bronchodilation, Beta 2 agonist, Antimuscarinic

## Abstract

Chronic obstructive pulmonary disease (COPD) represents a significant cause of global morbidity and mortality, with a substantial economic impact. Recent changes in the Global initiative for chronic Obstructive Lung Disease (GOLD) guidance refined the classification of patients for treatment using a combination of spirometry, assessment of symptoms, and/or frequency of exacerbations. The aim of treatment remains to reduce existing symptoms while decreasing the risk of future adverse health events. Long-acting bronchodilators are the mainstay of therapy due to their proven efficacy. GOLD guidelines recommend combining long-acting bronchodilators with differing mechanisms of action if the control of COPD is insufficient with monotherapy, and recent years have seen growing interest in the additional benefits that combination of long-acting muscarinic antagonists (LAMAs), typified by tiotropium, with long-acting β_2_-agonists (LABAs), such as formoterol and salmeterol. Most studies have examined free combinations of currently available LAMAs and LABAs, broadly showing a benefit in terms of lung function and other patient-reported outcomes, although evidence is limited at present. Several once- or twice-daily fixed-dose LAMA/LABA combinations are under development, most involving newly developed monotherapy components. This review outlines the existing data for LAMA/LABA combinations in the treatment of COPD, summarizes the ongoing trials, and considers the evidence required to inform the role of LAMA/LABA combinations in treatment of this disease.

## Introduction

Currently the fourth leading cause of death globally [1], chronic obstructive pulmonary disease (COPD) is a major cause of morbidity and mortality, projected to become the world’s third leading cause of mortality by 2020 [2]. Characterized by progressive airflow limitation, COPD also has a major economic impact, contributing to US$53.7 billion in related direct costs in 2008 in the US [3].

As an area of interest for physicians, and a logical progression from monotherapy for those seeking to further improve outcomes in patients with respiratory disease, the subject of combined bronchodilation with bronchodilators of differing modes of action has been discussed in the literature previously [4-8]. The aim of this review is to focus more specifically on the combination of long-acting muscarinic antagonists (LAMAs) and long-acting β_2_-agonists (LABAs) for the treatment of COPD, summarizing data from recent peer-reviewed publications and published abstracts.

## Evolving COPD guidelines

**Figure 1:**
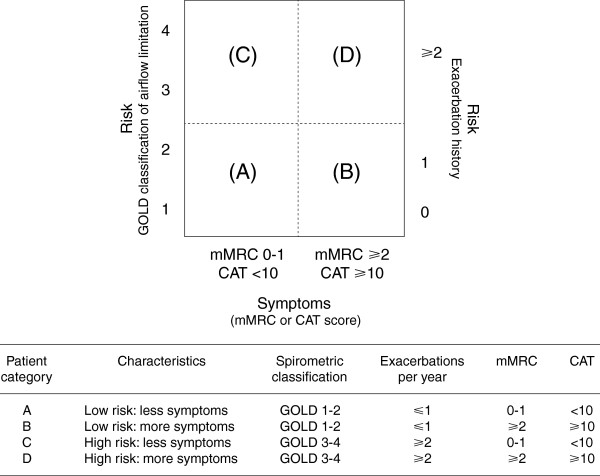
**Model of symptom/risk of evaluation of COPD **[9]**.** From the *Global Strategy for Diagnosis, Management and Prevention of COPD 2013*, Global initiative for chronic Obstructive Lung Disease (GOLD), http://www.goldcopd.org. COPD = chronic obstructive lung disease; GOLD = Global initiative for chronic Obstructive Lung Disease; mMRC = modified Medical Research Council; CAT = COPD Assessment Test.

2011 saw a substantial revision of the Global initiative for chronic Obstructive Lung Disease (GOLD) guidelines for diagnosis, management, and prevention of COPD, which has recently been updated [9]. Recommendations for treatment are no longer based primarily on categorization (“staging”) by spirometric assessment, but on categorization by existing symptoms (using validated modified Medical Research Council and COPD Assessment Test questionnaires) and risk (based on severity of airflow limitation and history of exacerbations). This approach acknowledges the importance of consideration of both short- and long-term outcomes when making treatment decisions (Figure 1) [9].

## Long-acting bronchodilators: the cornerstone of COPD maintenance therapy

Two key classes of bronchodilators have been developed in COPD: β_2_-agonists and muscarinic antagonists. Short-acting bronchodilators, such as ipratropium, albuterol, and metaproterenol, have formed the cornerstone of initial COPD therapy for the past two decades [9,10]. Subsequently, long-acting bronchodilators were developed. The twice-daily LABAs salmeterol and formoterol first became available for maintenance therapy of COPD more than 15 years ago, while the once-daily LAMA tiotropium has been available for 10 years and is the most widely prescribed maintenance monotherapy bronchodilator in COPD [11]. Inhaled bronchodilators, as monotherapy or in combination, remain the mainstay for patients in all categories (Table 1) [9]. Long-acting bronchodilators, such as tiotropium, formoterol, and salmeterol, are proven to provide long-term improvements in lung function, quality of life, and exacerbations in patients with COPD [12-14]. Long-acting bronchodilators (e.g., tiotropium, salmeterol) also reduce lung hyperinflation and dyspnea, and increase exercise endurance [15,16], although a recent review suggested further data are required on the benefits of long-acting bronchodilators on exercise tolerance [17]. There are a variety of long-acting bronchodilators available (Table 2) [18-34]. The once-daily LABA indacaterol, the once-daily LAMA glycopyrronium, and twice-daily LAMA aclidinium represent newer, recently licensed therapies, and there are also several once-daily LABAs and LAMAs in development, including olodaterol, vilanterol, and glycopyrrolate (Table 2).

**Table 1:** **GOLD guidelines (2013): pharmacologic therapies for stable COPD**^**a **^**[**[9]**]**

**Patient group**	**1st choice**	**2nd choice**	**Alternative**
A: GOLD 1-2	Short-acting anticholinergic prn **OR** SABA prn	LAMA **OR** LABA **OR** SABA and short-acting anticholinergic	Theophylline
Low risk of exacerbation, less symptoms			
B: GOLD 1-2	LAMA **OR** LABA	LAMA and LABA	SABA and/or short-acting anticholinergic
Low risk of exacerbation, more symptoms			Theophylline
C: GOLD 3-4	ICS + LABA **OR** LAMA	LAMA and LABA **OR** LAMA and PDE-4 inhibitor **OR** LABA and PDE-4 inhibitor	SABA and/or short-acting anticholinergic
High risk of exacerbation, less symptoms			Theophylline
D: GOLD 3-4	ICS + LABA and/or LAMA	ICS and LABA and LAMA **OR** ICS + LABA and PDE-4 inhibitor **OR** LAMA and LABA **OR** LAMA and PDE-4 inhibitor	Carbocysteine
High risk of exacerbation, more symptoms			SABA and/or short-acting anticholinergic
			Theophylline

From the *Global Strategy for Diagnosis, Management and Prevention of COPD 2013*, Global initiative for chronic Obstructive Lung Disease (GOLD), http://www.goldcopd.org.

^a^Medications in each box are mentioned in alphabetical order, and, therefore, not necessarily in order of preference.

prn = as needed; SABA = short-acting β_2_-agonist; LABA = long-acting β_2_-agonist, LAMA = laong-acting muscrannic antagonist; ICS = inhaled corticosteroid; PDE = phosphodiesterase.

**Table 2:** LABAs and LAMAs in development for combination therapies

**Therapy**	**LABA/LAMA**	**Manufacturer**	**Dosage**	**Time to onset**	**Trough FEV**_**1 **_**(difference from placebo)**
Formoterol	LABA	Merck^a^	Twice daily	5 min [26]	50–90 mL [26]
			4.5 μg (MDI) and 12 μg (DPI)		
Indacaterol	LABA	Novartis	Once daily 150 and 300 μg (EU) (DPI) [20]	5 min [22]	130–180 mL (p < 0.001) [22]
Indacaterol	LABA	Novartis	Once daily 75 μg (US) (DPI) [21]	5 min [22]	≥120 mL (p < 0.001) [23,33]
Olodaterol	LABA	Boehringer Ingelheim	Once daily 5 and 10 μg (Respimat®)	Not available	61–132 mL (p < 0.01) [24]
Vilanterol	LABA	GSK	Once daily 25 and 50 μg (DPI)	Median 6 min [25]	137–165 mL (p < 0.001) [25]
Aclidinium	LAMA	Almirall/Forest Laboratories	Twice daily 200–400 μg (DPI)	10–30 min [27,28]	86–124 mL (p < 0.0001) [29]
Glycopyrronium	LAMA	Novartis	Once daily 50 μg (DPI)	5 min [19,34]	91–108 mL (p < 0.001) [30,34]
Glycopyrrolate	LAMA	Pearl Therapeutics	Twice daily 36 μg (MDI)	5 min [19]	Statistically superior to placebo (p < 0.0001) [31]
GSK233705	LAMA	GSK	Twice daily 200 μg	Not available	130 mL (p < 0.001) [32]
Tiotropium	LAMA	Boehringer Ingelheim	Once daily 18 μg (DPI) and 5 μg (via SMI)	15 min [18]	120–150 mL (p < 0.001) [18]
Umeclidinium (GSK573719)	LAMA	GSK	Once daily	Not available	Not available

^a^Other companies are developing formoterol as part of a fixed-dose combination.

PubMed and European Respiratory Society and American Thoracic Society congresses were searched for abstracts relating to LAMA/LABA therapies known to be in development as part of a combination. As trials were carried out in different patient populations and there were differences in background medications allowed/utilized, no direct comparisons can be made.

DPI = dry powder inhaler; LABA = long-acting β_2_-agonist; LAMA = long-acting muscarinic antagonist; MDI = metered dose inhaler; SMI = Soft Mist™ Inhaler.

## Combination bronchodilation: rationale

Guidelines recommend combination therapy involving two long-acting bronchodilators with differing modes of action in patients whose COPD is not sufficiently controlled with monotherapy (Table 1) [9]. The mechanistic rationale for combination of a muscarinic antagonist and a β_2_-agonist has been reviewed in detail recently [35,36]; as such, we will only briefly discuss it here. Airway smooth muscle relaxation (leading to bronchodilation) can be achieved via two main routes: inhibition of acetylcholine signaling via muscarinic M_3_ receptors on airway smooth muscle with a muscarinic antagonist, or stimulation of β_2_-adrenoceptors with a β_2_-agonist [35,36]. Targeting these two mechanisms of bronchoconstriction, theoretically, has the potential to maximize the bronchodilator response without increasing the dose of either component, and helps to overcome the inter- and intra-patient variability in response to individual agents seen in COPD [35]. The interaction between the two systems has yet to be fully elucidated; however, β_2_-agonists can amplify the bronchial smooth muscle relaxation directly induced by the muscarinic antagonist by decreasing the release of acetylcholine via modulation of cholinergic neurotransmission. Additionally, muscarinic antagonists have been demonstrated to augment β_2_-agonist-stimulated bronchodilation by reducing the bronchoconstrictor effects of acetylcholine in preclinical models [35].

The rationale for improved bronchodilation has been tested in preclinical models with a specific investigational LAMA/LABA combination, tiotropium plus olodaterol, demonstrating synergistic effects on bronchoprotection *in vivo*[37,38]. Combination treatment in ovalbumin-induced bronchoconstriction in anesthetized guinea pigs has demonstrated improved bronchoprotection in a dose-dependent manner, with effective dose (ED) 50 values 10-fold lower than the ED50 of olodaterol alone (p < 0.05) [38]; similar results were reported versus tiotropium monotherapy in acetylcholine-induced bronchoconstriction in anesthetized dogs [37]. Indacaterol synergistically potentiates the effects of glycopyrronium to inhibit metacholine-induced contraction of airway smooth muscle *in vitro*[39]. However, specifically designed clinical studies are required to assess whether such synergistic effects can be observed with therapeutic doses in humans.

## Combination bronchodilation: existing evidence and ongoing studies

### Short-acting muscarinic antagonist plus short-acting β_2_-agonist

The concept of adding a muscarinic antagonist to a β_2_-agonist is by no means new. A fixed-dose combination (FDC) of the short-acting agents ipratropium and albuterol (Combivent®) and of fenoterol and ipratropium (Berodual®) clearly provides benefits over monotherapy with either component [40-42]. Additionally, dual bronchodilation with ipratropium and albuterol resulted in a consistently longer response and more patients achieved a pre-specified response level (12-15%) in forced expiratory volume in 1 second (FEV_1_) with the combination versus individual components (p < 0.05) [40,41,43-45], with an equivalent or improved safety profile.

### Free combinations of LAMA plus LABA

Relatively few studies have examined the combination of LAMAs and LABAs. Until recently, research focused on free combinations of existing therapies, and has demonstrated benefits on lung function and other outcomes (Table 3) [46-69].

**Table 3:** LABA and LAMA combinations: current evidence

**Combination (manufacturer)**	**Reference**	**Reported results**
**Free combinations**		
GSK233705: 20 or 50 μg BID; salmeterol: 50 μg BID	Beier et al. [61]	Larger mean increases from baseline trough FEV_1_ vs placebo with 20 μg GSK233705 + salmeterol (203 mL) and 50 μg GSK233705 + salmeterol (215 mL) vs monotherapy with tiotropium (101 mL) or salmeterol (118 mL)
Tiotropium: 18 μg QD; arformoterol: 15 μg BID	Tashkin et al. [60]	Greater improvement in FEV_1_ AUC_0-24_ from baseline with combination (0.22 L) vs monotherapy with either arformoterol (0.10 L) or tiotropium (0.08 L); p < 0.001
		Greater improvement in TDI with combination (3.1) vs monotherapy with either arformoterol (2.3; CI 0.03, 1.70) or tiotropium (1.8; CI 0.50, 2.20)
Tiotropium: 18 μg QD; formoterol: 20 μg BID	Hanania et al. [65]	FEV_1_ AUC_0-3_ greater with combination (1.57 L) vs tiotropium alone (1.38 L); p < 0.0001
		Reduced use of rescue medication vs tiotropium alone; p < 0.05
Tiotropium: 18 μg QD; formoterol: 20 μg BID	Tashkin et al. [56]	Greater FEV_1_ AUC_0-3_ with combination (1.52 L) vs tiotropium alone (1.34 L); p < 0.0001
		Greater improvement in TDI with combination than tiotropium alone (2.30 vs 0.16; mean difference of 1.80); 95% CI 0.86, 2.74, p < 0.0002
Tiotropium: 18 μg QD; formoterol: 12 μg BID	Tashkin et al. [55]	Greater improvement in FEV_1_ AUC_0-4_ from baseline with combination (0.34 L) vs tiotropium alone (0.17 L); p < 0.001
		Dyspnea significantly improved with combination at week 8 (1.86) vs tiotropium alone (1.01); p = 0.013
		Reduced use of rescue medication vs tiotropium alone; p < 0.04
Tiotropium: 18 μg QD; formoterol: 12 μg QD or BID	Terzano et al. [66]	Greater improvement in FEV_1_ with combination vs tiotropium alone at day 30 (0.16 L); p = 0.0001
		Improvement in dyspnea with combinations (2.32-2.61) vs tiotropium alone (1.0); p < 0.05
		Lower rescue medication use with combinations (0.71-0.80 puffs/day) vs tiotropium alone (2.14 puffs/day); p < 0.05
Tiotropium: 18 μg QD; formoterol: 12 μg QD or BID	van Noord et al. [47]	Greater average improvement in FEV_1_ AUC_0-24_ 0.16-0.20 L with combinations vs 0.08 L with tiotropium alone; p < 0.05
		Lower rescue medication use with combinations vs tiotropium alone; p < 0.01 (daily rescue medication use with tiotropium + formoterol QD or BID) and p < 0.05 (tiotropium + formoterol BID)
Tiotropium: 18 μg QD; formoterol: 12 μg QD or BID	van Noord et al. [49]	Average improvement in daytime (0.234 L) and night-time FEV_1_ (0.086 L) with combination vs monotherapy with tiotropium (p ≤ 0.001) or formoterol (p ≤ 0.01)
		Lower rescue medication use with combination (1.81 puffs/day) vs monotherapies (2.37-2.41 puffs/day); p < 0.01
Tiotropium: 18 μg QD; formoterol: 10 μg BID	Vogelmeier et al. [54]	Improvement in FEV_1_ 2 h post-dose after 24 weeks with combination vs formoterol alone (p = 0.044)
Tiotropium: 18 μg QD; indacaterol: 150 μg QD	Mahler et al. [50]	Greater increase in trough FEV_1_ from baseline with combination: 70–80 mL difference vs tiotropium alone (p < 0.001)
		Improved trough IC with combination vs tiotropium alone: 100–130 mL; p < 0.01
		Less use of albuterol as rescue medication with combination: reduction of 0.8–1.3 puffs/day from baseline vs tiotropium alone
Tiotropium: 18 μg QD; salmeterol: 50 μg BID	Aaron et al. [48]	No statistical improvement in lung function or hospitalization rates with combination compared to tiotropium monotherapy
Tiotropium: 18 μg QD; salmeterol: 50 μg BID	van Noord et al. [59]	Improved average FEV_1_ (0–24 h) with combination (0.142 L) vs monotherapy with either tiotropium (0.07 L) or salmeterol (0.045 L); p < 0.0001
		Combination associated with clinically relevant improvements in TDI focal score (p < 0.001)
**Fixed-dose combinations**		
Glycopyrrolate: 36 and 72 μg BID; formoterol: 9.6 μg BID (Pearl Therapeutics)	Reisner et al. [53]	Increase in FEV_1_ AUC_0–12_ on day 7 with combination compared to monotherapy with either of the components, tiotropium, and placebo (p < 0.0001)
Glycopyrrolate: 36 and 72 μg BID; formoterol: 9.6 μg BID (Pearl Therapeutics)	Reisner et al. [52]	Higher morning pre-trough and peak IC with combination vs placebo (p < 0.0005 and p < 0.005, respectively) or tiotropium monotherapy (p < 0.05 for all comparisons)
Glycopyrrolate: 36 and 72 μg BID; formoterol: 9.6 μg BID (Pearl Therapeutics)	Reisner et al. [62]	Similar metabolic and cardiac safety profile to tiotropium
Glycopyrronium: 50 μg QD; indacaterol: 300 μg QD (Novartis)	van Noord et al. [58]	Improved trough FEV_1_ with combination: 0.226 L difference in trough FEV_1_ vs placebo (p < 0.001)
		Greater peak FEV_1_ with combination (1.709 L) vs 300 μg indacaterol (1.579 L) and 600 μg indacaterol (1.573 L); p < 0.0001 for both comparisons
Glycopyrronium: 100 μg QD; indacaterol: 600 μg QD (Novartis)	Van de Maele et al. [57]	Increased trough FEV_1_ with combination (1.61 L) vs indacaterol monotherapy 300 μg (1.46 L); p < 0.05
Glycopyrronium: 50 μg QD; indacaterol: 110 μg QD (Novartis)	Bateman et al. [68]	Improved trough FEV_1_ with combination vs placebo (0.20 L mean difference), indacaterol (0.07 L), glycopyrronium (0.09 L), and tiotropium (0.08 L) monotherapy; p < 0.001
		Improved TDI score with combination vs placebo (mean difference 1.09); p < 0.001 and tiotropium (0.51 mean difference); p < 0.05
		Improved SGRQ score with combination vs tiotropium (-2.13 mean difference); p < 0.05
		Reduced use of rescue medication with combination vs monotherapies (-0.30 to -0.54 mean difference); p < 0.05
Glycopyrronium: 50 μg QD; indacaterol: 110 μg QD (Novartis)	Vogelmeier et al. [69]	Improvement in trough FEV_1_ with combination vs salmeterol/fluticasone (mean difference 0.103 L); p < 0.0001
		Improvements in TDI score with combination vs salmeterol/fluticasone (mean difference 0.76); p = 0.003
		Lower use of rescue medication with combination vs salmeterol/fluticasone (-0.39 puffs/day); p = 0.019)
Glycopyrronium: 50 μg QD; indacaterol: 110 μg QD (Novartis)	Dahl et al. [67]	Combination increased FEV_1_ and FVC vs placebo over a 52-week period; p < 0.001
Tiotropium: 5 μg QD; olodaterol: 2, 5, and 10 μg QD (Boehringer Ingelheim)	Maltais et al. [51]	Higher peak FEV_1_ for all doses of combination investigated vs tiotropium alone (p ≤ 0.05); higher trough FEV_1_ response with tiotropium + olodaterol 5/10 μg vs tiotropium alone (p = 0.034)
Tiotropium: 1.25, 2.5, and 5 μg QD; olodaterol: 5 and 10 μg QD (Boehringer Ingelheim)	Aalbers et al. [64]	Significant improvements in FEV_1_ for all doses of combination vs olodaterol alone, with evidence of a dose-dependent response
Umeclidinium (GSK573719): 500 μg QD; vilanterol: 25 μg QD (GSK)	Feldman et al. [63]	Adverse-event rate of 26%, with no single adverse event reported in >1 patient
		Combination similar to placebo in terms of cardiac parameters
		Greater change from baseline in trough FEV_1_ and FEV_1_ from 0–6 h post-dose with combination vs placebo

PubMed and relevant respiratory congresses were searched for abstracts relating to LAMA/LABA combination therapies.

BID = twice a day; FEV_1_ = forced expiratory volume in 1 second; QD = once a day; AUC = area under the curve; TDI = Transition Dyspnea Index; CI = confidence interval; IC = inspiratory capacity; SGRQ = St George’s Respiratory Questionnaire.

Following proof of concept in terms of a benefit on FEV_1_ and in patients with acute exacerbations [70-72], several randomized controlled trials have reported improved lung function for tiotropium plus formoterol versus tiotropium alone [54-56,60,65,73]. Some trials have also identified significant improvements in symptom scores [55,56,60] and reductions in rescue medication use [47,49,55,56,65]. A recent meta-analysis confirmed the benefits of tiotropium plus formoterol on average FEV_1_, trough FEV_1_, and Transition Dyspnea Index (TDI) [74] (Table 3), with initial reports also suggesting that the combination may provide statistically significant improvements in effort-induced dynamic hyperinflation and exercise tolerance [75].

Currently available data on tiotropium plus salmeterol are conflicting. Initial investigations indicated the benefits of tiotropium plus salmeterol versus either monotherapy alone, while suggesting co-administration of once-daily salmeterol plus tiotropium was inadvisable, due to the shorter duration of bronchodilation provided by salmeterol [76]. The Canadian Optimal Therapy of COPD trial investigated the impact of tiotropium plus placebo, tiotropium plus salmeterol, or tiotropium plus salmeterol/fluticasone on clinical outcomes in 449 patients with moderate to severe COPD [48]. Tiotropium plus salmeterol/fluticasone (but not tiotropium plus salmeterol) statistically improved lung function and quality of life, and, while no improvement in overall exacerbation rate was seen, reduced the number of hospitalizations for exacerbations compared to tiotropium plus placebo [48] (Table 3). A more recent study, however, demonstrated significant improvements in FEV_1_ with salmeterol once or twice daily plus tiotropium [59], and the combination was also associated with clinically significant improvements in TDI (Table 3). These inconclusive data suggest that further research is necessary to determine any advantage of salmeterol plus tiotropium. Initial investigation of other free combinations has also been reported (Table 3); tiotropium plus indacaterol has been demonstrated to improve lung function and inspiratory capacity, as well as providing a further reduction in use of rescue medication [50]. The LAMA GSK233705 twice daily plus salmeterol has also demonstrated significantly improved trough FEV_1_ from baseline compared to monotherapy [61]. Overall, these data broadly confirm that combination therapy has the potential to improve outcomes versus monotherapy and justify further research in this area.

### Fixed-dose combinations (FDCs)

FDCs of LAMAs and LABAs offer the potential of improved convenience and compliance over use of separate inhalers, and, during their development, the dose of each agent to be used in combination can be optimized.

A major challenge associated with development of an FDC is provision of improved bronchodilation over monotherapy components while balancing the associated adverse effects [77]. Dose-finding studies are required to establish minimal effective doses for each agent in the combination, as it cannot be assumed that these doses are the same as would be used in monotherapy. Regulatory bodies also require investigation into any pharmacodynamic or pharmacokinetic interactions that may occur between the constituents, along with evidence of the safety profile for the combination [77,78].

A number of FDCs are in development, with substantial clinical programs (Table 4), and some Phase II/III results are available (Table 3). In all cases, there is evidence of improved lung function parameters with the combinations versus monotherapy components. Glycopyrrolate/glycopyrronium is being developed as part of two combinations: the former plus formoterol as a twice-daily combination (Pearl Therapeutics) and the latter plus indacaterol as a once-daily combination (Novartis). Glycopyrrolate plus formoterol has recently reported improvements in FEV_1_ area under the curve from 0–12 hours (AUC_0-12_) versus monotherapy with glycopyrrolate, formoterol, or tiotropium [52], and in inspiratory capacity versus tiotropium monotherapy [53] (Table 3). Indacaterol is approved at doses of 150 and 300 μg in the EU, and 75 μg in the US [79]. Early studies investigated high doses of indacaterol in combination with glycopyrronium [57,58], but the recent SHINE, ILLUMINATE, and ENLIGHTEN studies have examined the effects of indacaterol (110 μg) plus glycopyrronium (50 μg) (Table 3). The SHINE study reported significantly greater improvements in trough FEV_1_ after 26 weeks’ treatment with the combination compared to monotherapy with indacaterol (mean difference 70 mL), glycopyrronium (90 mL), or tiotropium (80 mL) [68]. This study also demonstrated improvements in dyspnea (versus placebo and tiotropium), St George’s Respiratory Questionnaire (versus placebo), and reduced use of rescue medication (versus placebo and all monotherapies). The ILLUMINATE study has provided interesting information on the relative effects on lung function of a LABA/LAMA combination versus LABA/inhaled corticosteroids (ICS): significant, sustained, and clinically meaningful improvements in trough FEV_1_, peak FEV_1_, and FEV_1_ AUC_0-12_ with indacaterol plus glycopyrronium versus the LABA/ICS salmeterol/fluticasone (p < 0.001 for all comparisons) [69]. Mean treatment difference for indacaterol plus glycopyrronium versus salmeterol/fluticasone ranged from 103 mL (trough FEV_1_) to 155 mL (peak FEV_1_) [69]. Additionally, the longer-term ENLIGHTEN study reported significant improvements in lung function with the combination versus placebo sustained for 52 weeks, with no evidence of tachyphylaxis [67].

**Table 4:** LABA and LAMA combinations under investigation: ongoing trials

**LAMA**	**LABA**	**Trial numbers**	**Summary of ongoing Phase II/III studies**
Aclidinium	Formoterol	NCT01572792, NCT0143797, NCT01462942, NCT01437540, NCT01049360	4 Phase III studies and 1 Phase II study, examining the long-term efficacy and safety of 2 different doses of aclidinium + formoterol vs monotherapy with either component and placebo
Glycopyrrolate	Formoterol	NCT01587079, NCT01587079	2 Phase II studies (both recently completed), examining efficacy of the combination vs the monotherapy components and tiotropium
Glycopyrronium	Indacaterol	NCT01120691 (SPARK), NCT01202188 (GLEAM) (pivotal studies, both completed), NCT0171251, NCT01604278, NCT01727141, NCT01529632, NCT01709903	2 recently completed pivotal Phase III studies investigating efficacy and safety, exacerbations, exercise, and TDI, and 5 ongoing Phase III studies, examining safety and efficacy of combination vs placebo, monotherapy components, and salmeterol/fluticasone
Tiotropium	Olodaterol	NCT01431274, NCT01431287 (pivotal studies), NCT01525615, NCT01533922, NCT01533935, NCT01559116, NCT01536262	7 Phase III studies, investigating efficacy and safety of combination vs monotherapy components and effects on exercise
Umeclidinium (GSK573719)	Vilanterol	NCT01313637, NCT01316900, NCT01316913, NCT01313650 (pivotal studies, recently completed), NCT01716520, NCT01491802	6 Phase III studies (4 recently completed, 2 ongoing), focused primarily on efficacy, with other studies examining AE incidence, exercise endurance time, and exertional dyspnea

ClinicalTrials.gov was searched for Phase II and III trials of fixed-dose combinations of LAMA + LABA. Access date: 12/05/12.

LABA = long-acting β_2_-agonist; LAMA = long-acting muscarinic antagonist; AE = adverse event; TDI = Transition Dyspnea Index; FDA = Food and Drug Administration.

Clinical Phase II trials have investigated the optimal dosing for olodaterol added to a fixed dose of tiotropium [51] and for tiotropium added to a fixed dose of olodaterol [64]. Significant improvements in peak FEV_1_ were demonstrated with tiotropium/olodaterol 5/2 μg (p = 0.008), 5/5 μg (p = 0.012), and 5/10 μg (p < 0.0001) versus tiotropium monotherapy [51]. Significant improvements were also seen in trough FEV_1_ with tiotropium/olodaterol 5/10 μg versus tiotropium monotherapy (p = 0.034) [51], and with all doses of the combination tested versus olodaterol monotherapy, with evidence of dose ordering [64] (Table 3).

There is little information available from studies of aclidinium plus formoterol; only initial results of preclinical cardiac safety in beagle dogs have been reported [80].

Of the LAMA/LABA FDCs currently under investigation, tiotropium plus olodaterol, umeclidinium plus vilanterol, aclidinium plus formoterol, and glycopyrronium plus indacaterol have the largest Phase III programs, focusing on efficacy, safety, exacerbations, exercise, and dyspnea (Table 4). The development program for umeclidinium plus vilanterol involves four large pivotal trials, one large safety study, and two studies assessing exercise endurance, with two ongoing trials investigating lung function and efficacy. Phase III studies of glycopyrronium plus indacaterol will compare the combination with a range of comparators, including fluticasone/salmeterol combination and tiotropium, across a variety of end points (FEV_1_, exacerbations, TDI, and safety). The tiotropium plus olodaterol development program includes two main registration trials, three examining exercise and functional capacity, one examining long-term safety, and a comprehensive lung function trial.

Several of the LAMA/LABA FDCs in development will be delivered once daily (such as umeclidinium plus vilanterol and tiotropium plus olodaterol) while others will have twice-daily dosing (e.g., aclidinium plus formoterol). This diversity has the potential to increase the personalization of medication to individual needs; for instance, twice-daily combinations could be considered where patients suffer from night-time symptoms [81], while once-daily combinations may be prescribed to improve adherence [82].

## Delivery devices

Inhalation devices are intrinsically linked to the medication they deliver. As such, careful consideration of the most appropriate delivery device is crucial to optimal COPD therapy, along with adequate training and observation of patient technique.

The new devices currently in development for delivery of combination inhaled LAMA/LABAs for COPD fall into three main classes: pressurized metered dose inhalers (pMDIs), dry powder inhalers (DPIs), and a propellant-free Soft Mist™ Inhaler (SMI), each with its own advantages and disadvantages (Table 5) [83-93]. Poor inhaler technique is often a main cause of sub-optimal COPD management [83]; determining a suitable delivery device that each individual patient will be able to use can help to ensure adequate disease management. For instance, a spacer or holding chamber can be used to improve drug delivery via pMDIs in patients with poor coordination between actuation and inhalation. DPIs were designed to ease operation compared to pMDIs [94]. However, it remains important to provide patient training on the specific DPI, as confusion can arise regarding the differing loading techniques for individual devices (e.g., self-contained reservoirs or blister packs versus capsule insertion). Additionally, a higher inspiratory flow is required to operate this type of inhaler, meaning that it may not be appropriate for patients with very severe COPD [94,95]. The increased aerosol production time provided by an SMI (1.5 seconds compared to <0.5 seconds for most other inhalers) may be an advantage for patients with low inspiratory capacity or poor timing of inhalation to actuation, although teaching and observing the patient’s technique remains important [83].

**Table 5:** Devices for delivery of combination bronchodilators

**Type of delivery device**	**Description**	**Advantages**	**Disadvantages**	**Combination products in development**
pMDI	Drug is dissolved in propellant (generally HFA). When activated, a valve system releases a metered volume of propellant containing the medication	Can be more cost-effective than DPIs [84]	Can be difficult to synchronize actuation with inhalation [89,90]	Pearl inhaler for glycopyrrolate + formoterol (Pearl Therapeutics) [86]
		High fine-particle fraction, leading to better peripheral lung deposition	Evidence suggests fewer patients use pMDIs correctly without teaching [84]	
		Breath-actuated DPIs require no hand-lung coordination		
DPI	Drug is delivered in powder form on inspiration by the patient; de-aggregation of the powder and generation of the aerosol provided by the patient’s inspiratory effort	Activated by inhalation, avoiding synchronization issues [84]	Can be more expensive vs MDIs [84]	Breezhaler® for indacaterol + glycopyrronium (single-dose, Novartis) [87]
		Evidence suggests more patients have an accurate inhaler technique with DPIs without teaching [84]	Errors in inhaler technique can still occur [92]	Ellipta® Vilanterol (single-dose, GSK)
				Pressair® for aclidinium (multi-dose, Almirall) [88]
Soft Mist™ Inhaler	Delivers a metered dose of medication as a slow-moving “soft mist” through a unique nozzle system, which should lead to improved lung and reduced oropharyngeal deposition vs other types of inhaler [85,93]	Drug delivery is not dependent on patient’s inspiratory capacity or inspiratory effort, allowing consistent deposition regardless of lung function [85] and higher lung deposition in patients with poor inhaler technique [91]	Less clinical experience with this device; more safety data are required	Respimat® for tiotropium + olodaterol (Boehringer Ingelheim) [85]

pMDI = pressurized metered dose inhaler; HFA = hydrofluoroalkane; DPI = dry powder inhaler.

Non-adherence is a well-documented issue in COPD and is associated with detrimental effects on patient outcomes [96,97]. Irrespective of the device type, a single device with once-daily dosing may provide a significant advantage, potentially improving adherence and, therefore, patient outcomes. Adherence is inversely related to the number of medications patients take [96] and both treatment persistence and adherence in patients with COPD have been demonstrated to be lower in multiple long-acting inhaler users, compared to single long-acting inhaler users [98]. Additionally, a recent large retrospective study reported that adherence was significantly higher in those who initiated treatment with once-daily dosing versus more frequent dosing [82]. On the other hand, apart from the possible advantage of once- over twice-daily dosing on adherence, a twice-daily preparation has the potential advantage of greater improvement in night-time airflow and symptoms, an issue that has recently been reappraised [81].

## Consideration of potential adverse effects of LAMA/LABA combinations

The safety profiles of both LAMAs and LABAs are well understood. However, when combining two entities, it is important to understand both the similarities and differences in adverse events. Given that both muscarinic antagonists and β_2_-agonists can have a detrimental effect on the cardiovascular system [26,99], these adverse events need to be monitored carefully in development programs for combination products. Initial results suggest that the cardiovascular safety profile of glycopyrronium plus indacaterol is similar to placebo, with no clinically significant differences observed versus placebo [57,68], and, to date, no safety concerns have been identified with tiotropium plus olodaterol [51]. Free combinations of LAMA/LABA also seem well tolerated. No differences in blood pressure and pulse rate were observed with tiotropium plus salmeterol versus single-agent therapies [59] and the meta-analysis of tiotropium plus formoterol data reported no differences in cumulative incidence of adverse events for the combination (33.2%) versus tiotropium alone (36.0%), stating that drug-related severe adverse events and cardiac effects were relatively rare [74].

## Emerging therapeutic approaches

### Triple therapy

Triple therapy with LAMA plus LABA/ICS has also been investigated, demonstrating benefits over monotherapy on lung function [48,100-102]. Additionally, a pilot study of patients with advanced COPD reported that triple therapy combined with pulmonary rehabilitation provided a benefit in terms of lung function [103]. Some reports also suggest that triple therapy can provide additional benefits, such as reduction in exacerbation rate and mortality [104], although a recent systematic review concluded that further, longer-term studies are required to determine the benefits of tiotropium plus LABA and ICS [105], or the additional benefit of ICS on top of LAMA/LABA combinations.

### Dual muscarinic antagonist-β2-agonists (MABAs)

Another interesting concept is that of single molecules with muscarinic antagonist-β_2_-agonist (MABA) activity. While the prospect of combining muscarinic antagonism and β_2_-agonism into one entity is highly attractive, this is a new and challenging area of research [106]. The optimal MABA should be designed to achieve balanced, high potencies at both muscarinic and β_2_ receptors, for consistency. The furthest developed MABA is GSK961081, which has demonstrated effective bronchoprotection *in vivo* as proof of concept (NCT00478738) [106] and is currently in Phase IIb studies. MABAs provide a fixed ratio of muscarinic antagonist and β_2_-agonist activity at a cellular level, have a single pharmacokinetic profile, and deliver a fixed ratio of muscarinic antagonist and β_2_-agonist to the whole lung, simplifying both combination delivery device and clinical development programs [4,5,107]. These properties suggest that MABAs have the potential to act as a useful platform for triple therapy with an anti-inflammatory agent.

### Novel bronchodilators

A number of new bronchodilators with novel modes of action are currently in early stages of development, including K^+^ channel openers, Rho kinase inhibitors, and analogs of vasoactive intestinal peptide; these have been reviewed more extensively elsewhere [7]. Given the substantial evidence supporting a role of Rho kinase in bronchoconstriction [108], Rho kinase inhibitors such as Y-27632, Y-30141, Y-30694, and fasudil may currently hold the most promise, demonstrating smooth muscle relaxant properties *in vitro*[7]. Once further evidence of efficacy and safety is available, these newer classes might be used in combination with more conventional bronchodilators, leading to additional therapeutic options and increased potential for individualized medication.

## Conclusions

In summary, there is considerable evidence and guidance to support use of the combination of a LAMA and a LABA in COPD, a number of free LAMA and LABA combinations have been studied, and several LABA/LAMA FDCs are under development.

Although there is a clear rationale for the use of LAMA and LABA combinations, a recent treatment regimen analysis for patients with COPD (based on prescription and medical claims) suggests that currently this free combination is utilized much less frequently than LAMA monotherapy (tiotropium), LABA/ICS combinations, and the triple combination of LAMA plus LABA/ICS in clinical practice (Table 6). These data show that regardless of the presence or absence of comorbid asthma, LABA/ICS is the most widely used of these regimens, followed by tiotropium monotherapy. Interestingly, triple therapy is already a substantially used therapeutic option (usage approximately 60% of that of tiotropium monotherapy). LABA/ICS is more frequently used in patients with comorbid asthma and COPD [109]. While this practice is to be expected, it is likely that the perceived overlap between asthma and COPD is exaggerated due to the common misconception that a significant bronchodilator response in patients with COPD implies the coexistence of an asthmatic component.

**Table 6:** **Analysis of medical and prescription claims, May 2011-April 2012**[109]

	**Mean monthly patient volume**^**a**^		
**Regimen**	**Total COPD**	**COPD only**	**COPD + asthma**
Tiotropium monotherapy	310,423	279,530	30,893
ICS/LABA	451,019	360,760	90,259
LABA + LAMA	5,888	5,479	410
Tiotropium + ICS/LABA	193,137	170,063	23,074

^a^Data from IMS Health, LifeLink solutions, calculated from US CMS-1500 medical claims and NCPDP prescription claims during period May 2011-April 2012.

COPD = chronic obstructive pulmonary disease; ICS = inhaled corticosteroid; LABA = long-acting β_2_-agonist; LAMA = long-acting muscarinic antagonist.

The very limited clinical use of LAMA/LABA combination therapy indicated by these data is perhaps surprising, given the current evidence base and GOLD guidance. However, the evidence from ongoing programs will be much more substantial and the future availability of LAMA/LABA FDCs should make this therapeutic option more convenient than separate administration by two inhalers (typically with different dosing schedules), currently the only option.

The rising number of both single-agent and combination therapies for COPD increases the number of treatment options, and, hence, makes treatment choice more complex. Further studies should be designed to provide substantial evidence for future guideline recommendations. In particular, investigation into the optimal sequencing of monotherapies and combination bronchodilators would be of use to determine where LAMA/LABA FDC therapy may be positioned in the treatment algorithm for COPD. Furthermore, examination of the benefits of using dual bronchodilation as initial maintenance therapy would be of interest, along with which groups of patients may benefit from this approach.

There is a growing appreciation of the benefits of bronchodilation beyond lung function, such as exacerbations [110], patient-reported outcomes, exercise tolerance [111] and exercise capacity, and daily activities [16,112]. Trials of LABA/LAMA FDCs should assess this wider range of outcomes in order to more fully understand the broader benefits of increased bronchodilation on a patient’s life as a whole.

## Abbreviations

AUC0-12: Area under the curve from 0–12 hours; COPD: Chronic obstructive pulmonary disease; DPI: Dry powder inhaler; ED: Effective dose; FDC: Fixed-dose combination; FEV1: Forced expiratory volume in 1 second; GOLD: Global initiative for chronic Obstructive Lung Disease; ICS: Inhaled corticosteroid; LABA: Long-acting β_2_-agonist; LAMA: Long-acting muscarinic antagonist; MABA: Muscarinic antagonist-β_2_-agonist; pMDI: Pressurized metered dose inhaler; SMI: Soft Mist™ Inhaler; TDI: Transition Dyspnea Index.

## Competing interests

DPT has received grant support from Boehringer Ingelheim, Pearl Therapeutics, Novartis, Forest, and GlaxoSmithKline, speaking fees from Boehringer Ingelheim, Novartis, and Forest, and undertaken consultancy for Pearl Therapeutics, Novartis, and Theravance.

GTF has received grant support from Boehringer Ingelheim, GlaxoSmithKline, Pearl Therapeutics, Novartis, Forest, and Pfizer, speaking fees from Boehringer Ingelheim, GlaxoSmithKline, Astra Zeneca, Novartis, and Forest, and has acted as an advisor to Boehringer Ingelheim, GlaxoSmithKline, Astra Zeneca, Pearl Therapeutics, Forest, and Novartis.

## Authors’ contributions

DPT and GTF were responsible for the concept and design of the paper, preparation and revision of the draft, and take full responsibility for the final version of this manuscript.
